# Hereditary Spastic Paraplegia: From Genes, Cells and Networks to Novel Pathways for Drug Discovery

**DOI:** 10.3390/brainsci11030403

**Published:** 2021-03-22

**Authors:** Alan Mackay-Sim

**Affiliations:** Griffith Institute for Drug Discovery, Griffith University, Brisbane, QLD 4111, Australia; a.mackay-sim@griffith.edu.au

**Keywords:** neurodegeneration, motor neuron disease, spastic paraplegia, endoplasmic reticulum, protein-protein interaction network

## Abstract

Hereditary spastic paraplegia (HSP) is a diverse group of Mendelian genetic disorders affecting the upper motor neurons, specifically degeneration of their distal axons in the corticospinal tract. Currently, there are 80 genes or genomic loci (genomic regions for which the causative gene has not been identified) associated with HSP diagnosis. HSP is therefore genetically very heterogeneous. Finding treatments for the HSPs is a daunting task: a rare disease made rarer by so many causative genes and many potential mutations in those genes in individual patients. Personalized medicine through genetic correction may be possible, but impractical as a generalized treatment strategy. The ideal treatments would be small molecules that are effective for people with different causative mutations. This requires identification of disease-associated cell dysfunctions shared across genotypes despite the large number of HSP genes that suggest a wide diversity of molecular and cellular mechanisms. This review highlights the shared dysfunctional phenotypes in patient-derived cells from patients with different causative mutations and uses bioinformatic analyses of the HSP genes to identify novel cell functions as potential targets for future drug treatments for multiple genotypes.

## 1. Introduction

Motor neuron diseases fall along a continuum of those affecting the lower motor neurons (the motor neurons in the spinal cord controlling muscles) to those affecting the upper motor neurons (the neurons in the motor cortex controlling the lower motor neurons with some diseases affecting both lower and upper neurons to a greater of lesser degree [1]. All HSPs affect the upper motor neurons, but many also show lower motor neuron signs [2,3].

Hereditary spastic paraplegias (HSPs) are a diverse collection of Mendelian genetic disorders linked together by the clinical observations of lower limb weakness and spasticity and bladder problems. The HSP genes and their clinical manifestations were recently comprehensively reviewed [2,3,4,5,6,7]. HSPs may be autosomal dominant (AD), autosomal recessive (AR), X-linked, and in the mitochondrial genome. HSP clinical classifications are designated “SPG1-80”, with many of the causative genes identified (Table 1). Collectively, HSP is rare, affecting about 1–5 persons per 100,000 with mutations in *SPAST* comprising about 40% of cases. HSP prevalence is variable around the world, estimated overall from a metanalysis as 1.8/100,000 [8], but regions differ, especially in AR cases (e.g., AR in Norway, 0.6%/100,000 AR; in Tunisia, 5.7/100,000 [9]. In Germany, overall prevalence is 2/100,000 [7]. Many HSP genes are found in only one or two consanguineous families.

Clinically HSPs are often categorized as “pure” (also called “uncomplicated or simple”) versus “complicated” (also called “complex”). Pure forms of HSP have signs and symptoms limited to the defining diagnosis of HSP, arising from degeneration of the corticospinal tract, whereas complicated forms have a variable set of other signs and symptoms that indicate widespread dysfunctions of many brain regions and many neuronal types: ataxia, seizures, intellectual disability, dementia, muscle atrophy, extrapyramidal disturbance, and peripheral neuropathy often specific to the genotype [4,5,6,7]. These non-pyramidal signs can make differential diagnosis difficult because they overlap with other neurodegenerative diseases (e.g., spastic ataxias, spinocerebellar ataxias, PSEN1-related disorders, inherited dementias, amyotrophic lateral sclerosis, spinal muscular atrophy) [2]. SPG4/*SPAST,* the most common form of the disease, is classified as pure, with loss of corticospinal tracts affecting motor functions in the lower limbs as well as bladder function. Dementia is observed in advanced disease, suggesting that a complicated etiology can emerge with advancing age and severity, even in a “pure” disease. A recent study of 608 HSP patients showed that complicating symptoms (dysphagia, cognitive impairment, extrapyramidal involvement, peripheral motor involvement, dysarthria, ataxia, and psychosis) were associated with more severe disease [7]. 

With the recent advances in gene editing, there is much said about the benefits of “personalized medicine” in which patient genotype is targeted for correction. For HSP, generally this is an unlikely solution since such “personalized medicine” would require therapies aimed at each of the 80 genes, each of which could contain one of many causative mutations in an individual and two in compound heterozygotic autosomal recessive individuals. The alternative is to look for therapies that can be applied to multiple genotypes based on molecular mechanisms shared among the HSP genotypes that link them to specific cellular networks, pathways, and disease-causative dysfunctions. That possibility is the focus of this review.

## 2. Pathological Phenotypes in White Matter Tracts in Hereditary Spastic Paraplegias (HSP) Patients

That HSP is a disease predominantly of upper neurons is confirmed in the radiological evidence, but HSP also affects the white matter tracts more broadly. The most common neuroanatomical changes occurring in HSP are loss of axons in the corticospinal tract (70% of all studies) and corpus callosum (80% of all studies) [4,14,15,16,17,18,19,20,21,22]. Loss or damage to axons in the corticospinal tract and corpus callosum are consistent with the motor symptoms of the disease, although white matter disturbances are not confined to these regions. The whole brain, frontal and temporal lobes, cerebellum, and other regions also show imaging changes. Numerous radiological measures reflect these changes and correlate strongly with disease severity and duration [15,16,19,21,23]. MRIs of the cervical spinal cord revealed genotype-specific differences [24,25,26]. In SPG4 and SPG11, there was both grey and white matter loss including in the corticospinal tract, with SPG11 patients having signs of damage in the anterior horns, not seen in SPG4 patients [25]. There were no grey or white matter abnormalities in some SPG7 or SPG3A patients [25], although atrophy was observed in other SPG3A patients as well as in SPG4, SPG6, and SPG8 patients [26]. There were also genotype-associated patterns of grey and white matter loss in the brain [24]. SPG4 patients had no cerebral or cerebellar abnormalities, but they had volumetric reductions in thalamus, caudate, red nucleus, corpus callosum, and some other regions [24], suggesting neural substrates of cognitive decline in SPG4 [27]. Patients with SPG7 had white matter changes around the corpus callosum, no cerebral cortex thinning, but significant and widespread cerebellar atrophy [24] that correlates with the frequent ataxia observed in these patients. SPG8 patients had another pattern of abnormalities with symmetrical cerebral cortical thinning at pre- and post-central gyri with reduced volume of the pallida [24]. SPG11 patients had the most widespread abnormalities: cerebral cortex, cerebral white matter, basal ganglia, deep cerebellar nuclei [24]. There were no abnormalities in grey or white matter in SPG3A patients including cerebral or cerebellar abnormalities [24].

These numerous observations indicate systemic neurodegeneration that is not restricted to the cortical motor neurons projecting to the distal spinal cord: even patients with “pure” forms can have loss of white matter tracts more often seen in more “complicated’ forms. Nonetheless, axon degeneration in the upper motor neurons projecting to the lower limbs is a key diagnostic feature in all HSP diagnoses. There is no evidence for death of the cortical motor neurons. Hence, axon degeneration will be key to understanding the genetic and cellular mechanisms shared across the many genetic forms of HSP. Indirect evidence for this can be gleaned from animal models and in cell lines in vitro using the molecular genetics approach of under- and over-expression of genes of interest.

## 3. Pathological Phenotypes in HSP Patient-Derived Cells

Cellular phenotypes have been most well studied in cells derived from individuals with SPG4/*SPAST*. SPG4 is a pure form of HSP, autosomal dominant and the most common form of HSP. Patient-derived cells have also been studied in SPG3A, SPG5, SPG7, SPG11, SPG13, and SPG15, covering the rest of the more common diagnoses and including pure and complicated forms. Most investigations discussed below use neurons generated from induced pluripotent stem cells and neural stem cells, although some use fibroblasts and lymphoblastoid cells.

### 3.1. SPG4/SPAST

SPG4/*SPAST* encodes the protein spastin, a microtubule severing protein that cuts stable, acetylated microtubules. As an unbiased first step in understanding the cellular pathology of *SPAST* mutations, we investigated genome-wide gene expression in olfactory neural stem (ONS) cells from nine SPG4 patients with six different mutations in *SPAST,* comparing them with ONS cells from ten healthy controls [28]. Surprisingly, 57% of the 10,000 expressed genes were significantly differentially expressed in patient cells. This level of disease-associated expression has not been reported previously in any disease and is certainly much higher than we have seen in ONS cells from people with schizophrenia, ataxia telangiectasia, or Parkinson’s disease [29,30,31]. Despite the massive differential gene expression in patient cells, they had a similar immunophenotype to control cells, a similar rate of cell proliferation and similar performance in six assays of cell metabolism [28]. Among the differentially expressed genes, 118/298 microtubule function genes were differentially expressed including 10 tubulins (which form microtubules) and 15 kinesins (which carry vesicles and organelles on microtubules) [28]. Gene Ontology (GO) analysis identified 11 microtubule-associated functions that were significantly over-represented in the differentially expressed genes, mostly functions concerning microtubule dynamics [28]. Among the individual genes, STMN1 was overexpressed in patient cells and contributed to 7/11 GO functions. STMN1 codes for stathmin, a microtubule-depolymerizing protein. Protein analysis revealed a 50% loss of spastin in patient cells, a similar loss of acetylated α-tubulin (the enzymic target of spastin) and an increase in stathmin [28]. Quantitative microscopy demonstrated that patient cells were significantly smaller than control cells, with less acetylated α-tubulin throughout the cell. The distribution of mitochondria and peroxisomes were altered: fewer peripherally located mitochondria and more peripherally located peroxisomes, suggesting that anterograde transport of mitochondria and retrograde transport of peroxisomes may be affected in patient cells. Live cell imaging revealed that peroxisomes in patient ONS cells moved more slowly than in control ONS cells [28]. In axon-like processes of differentiated ONS cells, there were fewer peroxisomes and slower rates of microtubule-dependent transport of peroxisomes with a reduction in the relative numbers of peroxisomes moving retrogradely [32]. Patient ONS cells were under oxidative stress at baseline and were more sensitive to oxidative stress induced by treatment with hydrogen peroxide [32].

Patient-derived forebrain glutamatergic neurons were generated from induced pluripotent stem (iPS) cells from three SPG4/SPAST patients and three healthy controls [33]. Like patient-derived ONS cells, patient-derived neurons had 50% spastin and 50% acetylated α-tubulin, a significant reduction in the speed of axonal peroxisome transport and fewer peroxisomes present in the axons [33]. Patient neurons were more sensitive to hydrogen peroxide-induced oxidative stress as shown by increased axon degeneration [33]. Patient-derived neurons have abnormal swellings in their axons composed of acetylated α-tubulin, which could interfere with microtubule-based organelle transport [33]. Axon swellings are a histopathologic hallmark of HSP in the spinal cords of patients and in a mouse model of HSP [34]. Axon swellings were also more frequent in neurons generated from other SPG4/SPAST HSP patients [35,36,37] and were associated with reduced transport of mitochondria including reduced retrograde transport [35,36,37]. Finally, another phenotype in SPG4/SPAST patient-derived forebrain neurons is a reduced outgrowth of neurites and increased size of growth cones [36,37].

In summary, the SPG4/SPAST cell phenotype can be characterized as smaller cells with less spastin, less acetylated α-tubulin, impaired microtubule-based transport of mitochondria and peroxisomes, and increased oxidative stress, with greater sensitivity to hydrogen peroxide. In neurons, this leads to axonal swellings, impaired neurite growth, and axon degeneration.

### 3.2. SPG7

SPG7 HSP is the most common autosomal recessive form leading to complicated HSP. *SPG7* encodes paraplegin, a zinc metalloproteinase localized to the inner mitochondrial membrane. It is a component of the mitochondrial permeability transition pore complex and is required for the efficient assembly of mitochondrial complex I [38]. Mutations in *SPG7* lead to mitochondrial dysfunctions [6,38]. Muscle tissue from SPG7 patients exhibits defects in oxidative phosphorylation [39] and Spg7 null mice have axonal swellings with accumulated mitochondria and neurofilaments, indicating that both mitochondrial function and axonal transport are impaired [40].

We examined mitochondrial structure and function in patient-derived ONS cells from patients with compound heterozygous mutations in *SPG7* and compared them with cells from patients with SPAST mutations and healthy controls [41]. All cells expressed paraplegin including the SPG7 patient cells. The SPG7 cells, but not the SPAST cells, had reduced mitochondrial morphology (reduced length and interconnectivity), reduced mitochondrial mass, smaller mitochondria, reduced mitochondrial membrane potential, impaired oxidative phosphorylation, reduced ATP production and content, accompanied by increased oxidative stress, compared to SPAST and control cells [41]. SPG7 patient cells also proliferated at a slower rate than control cells [33], in contrast to SPAST patient cells [28].

These results confirm and extend the observations that SPG7 mutations affect mitochondrial function and demonstrate that SPG4 mutations do not have the same severe effects on mitochondrial structure and function in ONS cells, being very similar to control cells in most assays [41]. There are no published SPG7 HSP patient-derived neurons with which to confirm these cell phenotypes. Fibroblasts from two SPG7 patients had mild and heterogeneous impairment of oxidative phosphorylation; fibroblasts from another patient did not [42]. Fibroblasts from two patients showed increased susceptibility to reactive oxygen species and evidence of impaired complex I activity [43].

The SPG7 cell phenotype can therefore be characterized as having significantly impaired mitochondrial morphology and functions including reduced oxidative phosphorylation. This phenotype is specific to SPG7 insofar as it was absent in SPG4 [41].

### 3.3. Other Genotypes

SPG3A/ATL1 HSP is an early onset, autosomal dominant, pure HSP. ATL1 encodes Atlastin. Atlastin binds to spastin [44] and is associated with Golgi and endoplasmic reticulum [45]. Neurons generated from iPS cells of one SPG3A/ATL1 patient had fewer, shorter, and less branched neurites and mitochondrial transport impairments (fewer motile mitochondria, less anterograde and retrograde transport) [46]. Interestingly, the impaired trafficking of mitochondria in SPG3A was not accompanied by loss of spastin [46].

SPG5/CYP7B1 HSP is a heterogeneous autosomal recessive, complicated HSP. SPG5 encodes the CYP7B1 gene, a paralog of CYP7A1, both of which are involved in cholesterol metabolism [47]. CYP7B1 converts 27-hydroxycholesterol into 5-cholesten-3β,7α,27-triol [48]. Mice with Cyp7B1 knocked out had raised levels of 27-hydroxycholesterol and 25-hydroxycholesterol [47,49], whose plasma and cerebrospinal fluid levels were highly raised in SPG5 HSP patients [50].

SPG11 HSP causes the most frequent autosomal recessive, complex HSP, and juvenile onset amyotrophic lateral sclerosis [51]. SPG11 encodes spatacsin. Neural precursor cells were generated from three patients with SPG11 mutations [51]. These had reduced proliferation and neurogenesis and downregulation of cell cycle genes [51]. SPG11 patient neurons derived from iPS cells had impaired transport of synaptic vesicles (fewer motile vesicles, less retrograde movement) [52] and impaired neurite growth (fewer, shorter, less branched) [52,53].

SPG13/HSPD1 HSP is a late-onset, autosomal dominant, pure HSP. HSPD1 encodes a mitochondrial chaperonin protein (HSP60) that is essential for the correct folding of mitochondrial proteins [54]. In SPG13 patient-derived fibroblasts and lymphoblastoid cells lines, there was no difference from controls in viability or sensitivity to hydrogen peroxide or differences between patients and controls in mitochondrial membrane potential [54]. They were therefore different from both SPG4 and SPG7 ONS cells.

SPG15/ZFYVE26 and SPG48/AP5Z1 are autosomal recessive genes that cause complicated HSP. ZFYVE26 encodes spastizin, which is a binding partner of AP5Z1 protein [55]. SPG15 and SPG48 patient-derived neurons generated from iPS cells from individuals with mutations in each gene had shorter neurites and axon swellings [55]. Mitochondrial morphology was affected with both genotypes showing reduced mitochondrial length and reduced mitochondrial densities in axons. They also had reduced mitochondrial membrane potentials [55].

A HSP cell phenotype emerges from this consideration of patient-derived cells of these HSP genes. SPG4, SPG15, and SPG48 patient-derived neurons have axon swellings. SPG3A, SPG4, SPG11, SPG15, and SPG48 have impaired neurite outgrowth. SPG3A, SPG4, and SPG11 have impaired organelle transport (peroxisomes, mitochondria, synaptic vesicles). Impaired mitochondrial functions were observed in neurons, ONS cells, or fibroblasts in SPG7, SPG15, and SPG48. Involvement in cholesterol metabolism and transport were seen in SPG4, SPG5, and SPG11. This summary must be incomplete because not all functions have been assayed in all genotypes. Most of these studies for each genotype are based on one or two patients (often with the same mutation) and one or two controls, so the applicability of the findings to other mutations in the same genotype needs confirmation. Nevertheless, the evidence of shared phenotypes across different genotypes is quite compelling for the concept that new drugs might be found that treat more than one genotype.

Taken together, the clinical definitions, shared symptoms and signs of the various HSP genotypes and the overlapping cell dysfunctions in patient-derived cells with different HSP genotypes, all suggest that HSP causative genes are interlinked in networks and biochemical pathways that regulate a shared cell biology. One way to identify these networks and pathways is through bioinformatic tools and databases. An example of this is the STRING database of known and predicted protein–protein interactions (PPIs) including “direct (physical) and indirect (functional) associations” [56]. (https://string-db.org; accessed on 27 January 2021).

## 4. Highly Connected Protein–Protein Interaction Networks of HSP Genes

A key question is whether HSP genes normally act together to regulate shared functions. Does each gene act independently to alter cell phenotype or do they act together in a regulatory network? A regulatory network would open the possibility for new therapies targeted at ameliorating network dysfunction across genotypes. This approach does not address the genetic mutation directly, but focuses on the downstream functional consequences of the mutation. An example of this is the use of tubulin-binding drugs to moderate the effects of SPAST mutations on organelle transport by affecting microtubule stabilization independently of SPAST [32]. To aid understanding in the discussion below, “HSP gene” is used in reference to the proteins, rather than the formal protein name.

The STRING database (version 11.0b) and Gene Ontology (GO) analysis were used to ask whether human HSP genes were interconnected in PPI networks and to identify those functions regulated by HSP PPI networks. The default settings of the analyses were used except that the interaction sources were limited to “text mining, experiments, databases, and co-expression” [56]. STRING represents the proteins as “nodes” and their interactions as lines connecting the nodes (“edges”). The program calculates an enrichment p-value comparing the number of edges expected for sets of proteins selected at random compared to the observed number of edges in the network. Enrichment values were similarly calculated for the GO analyses using false discovery rate (FDR). Network interactions were limited either to direct interactions between the proteins of interest or set for a maximum of 10 interactions in the first and second shell. These latter are binding partners of directly interacting partners.

STRING analyses were performed on four sets of HSP genes: the set of the six highest prevalence pure SPG classifications, a larger set of 15 pure SPG classifications including rarer types, a set of 42 HSP genes with complicated SPG classifications, and a set of 57 HSP genes that includes pure and complicated SPG classifications (Table 1). The rationale for this step-wise design was that analysis of pure forms may identify networks related to the defining clinical signs and symptoms of SPG diagnosis while the larger network may identify networks related to the many different effects of the complications induced by the majority of HSP genes. For each set of genes, two networks were identified: the network of direct interactions between the proteins and the network of indirect interactions extended to the second shell of interactions. Discussions of each network were limited to the top 10 most highly significant GO categories of Biological Process and Cellular Component (Table 2 and Table 3). Full statistical results of the GO analysis are presented in the Supplementary Tables S1–S8.

### 4.1. Networks of the Highest Prevalence Pure SPGs (Six Genes)

**Figure 1:**
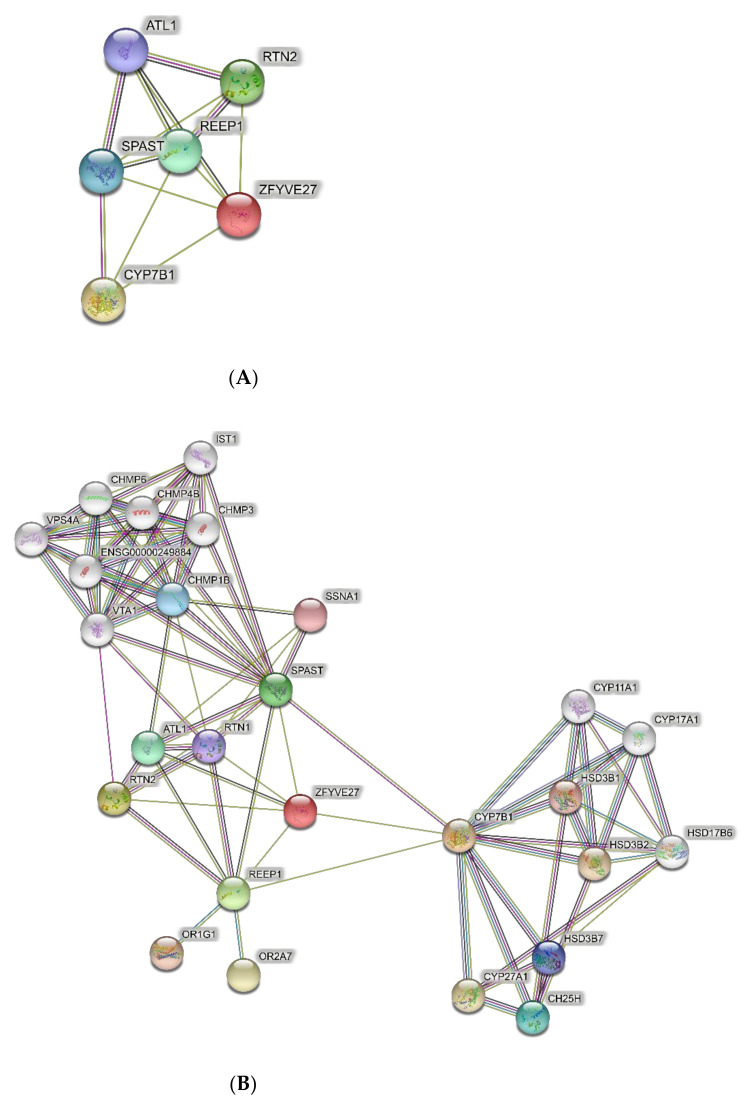
Predicted protein–protein interactions (PPI) networks for the six genes of the most common SPGs. (**A**) Direct interactions. (**B**) Second shell interactions.

Predicted PPI networks were based on the following genes, which are responsible for the majority of SPG cases: ATL1 (SPG3), SPAST (SPG4), CYP7B1 (SPG5A), RTN2 (SPG12), REEP1 (SPG31), and ZFYVE27 (SPG33). The network of direct interactions between these proteins is shown in Figure 1A. The PPI enrichment p-value for this network was 6.54 × 10^−13^, indicating a very highly interconnected network. Extending this network to the second shell revealed two tight secondary clusters of related proteins (Figure 1B; PPI enrichment *p* < 1.0 × 10^−16^): the ESCRT III pathway (CHMP and VPS proteins), which is central to the formation and sorting of endosomal cargo proteins bound for the lysosomes in multivesicular bodies [57], and a cluster associated with CYP17B1 that regulates steroid biosynthesis including in the brain [58,59].

### 4.2. Networks of Pure SPGs (15 Genes)

**Figure 2:**
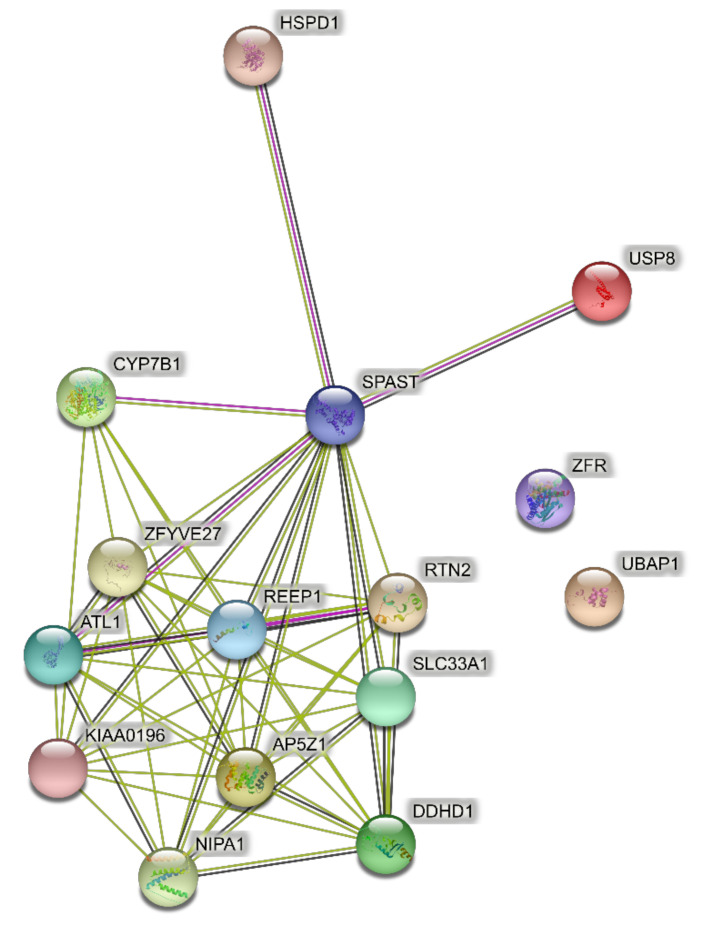
Predicted PPI networks for direct interactions among 15 genes of pure SPGs.

**Figure 3:**
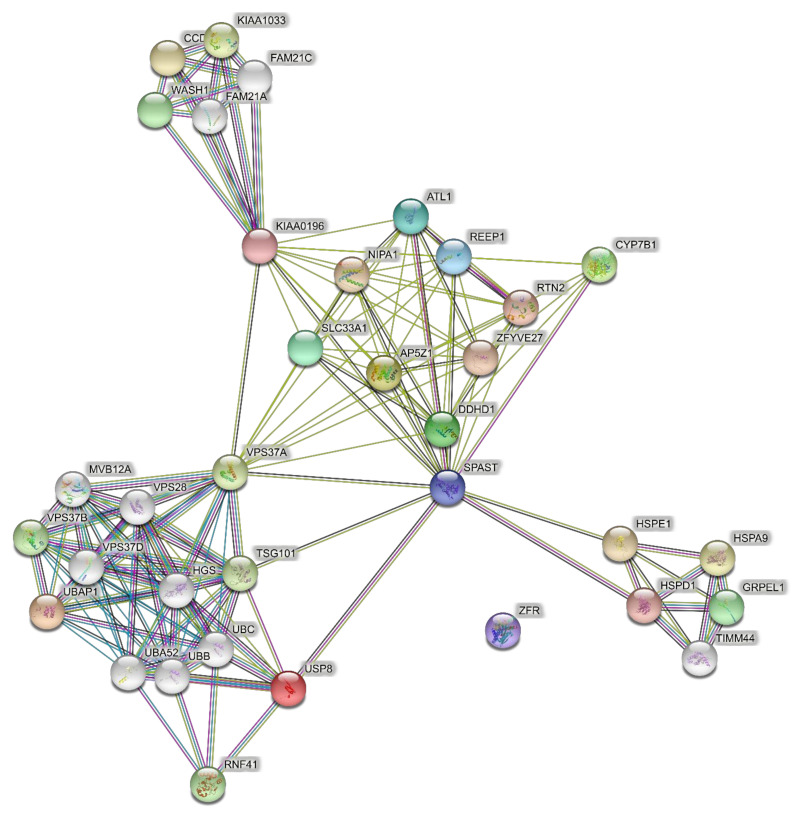
Predicted PPI networks for second shell interactions among 15 genes of pure SPGs.

The input to PPI networks was extended to a larger set of 15 HSP genes responsible for pure SPG classifications (Table 1) including the six in the analysis above. The network of direct interactions between these proteins is shown in Figure 2 (PPI enrichment *p* = 1.0 × 10^16^). Of the 15 genes, 12 were linked in an interconnected network, with SPAST directly interacting with most of them, showing its centrality to the disease process (Figure 2). The second shell revealed three secondary clusters of related proteins (Figure 3; PPI enrichment *p* < 1.0 × 10^−16^): the ESCRT I pathway (bottom left cluster, VPS proteins), the WASH complex (top left cluster), and protein chaperones (bottom right cluster).

### 4.3. Networks of the Set of Complicated SPGs (42 Genes)

**Figure 4:**
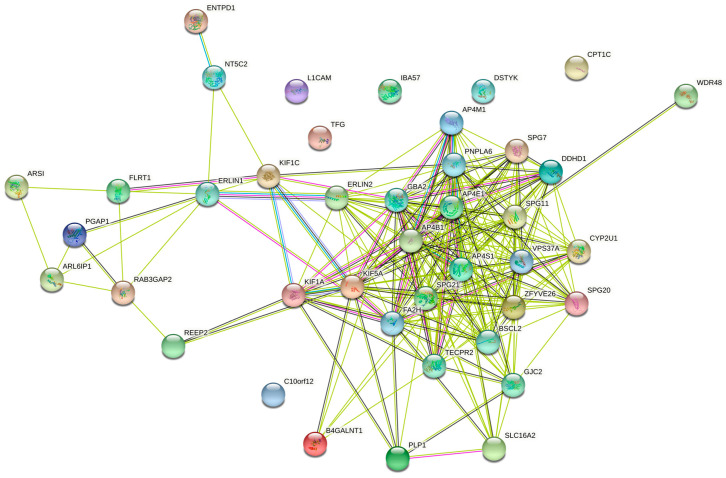
Predicted PPI networks direct interactions among 42 genes of complicated SPGs.

**Figure 5:**
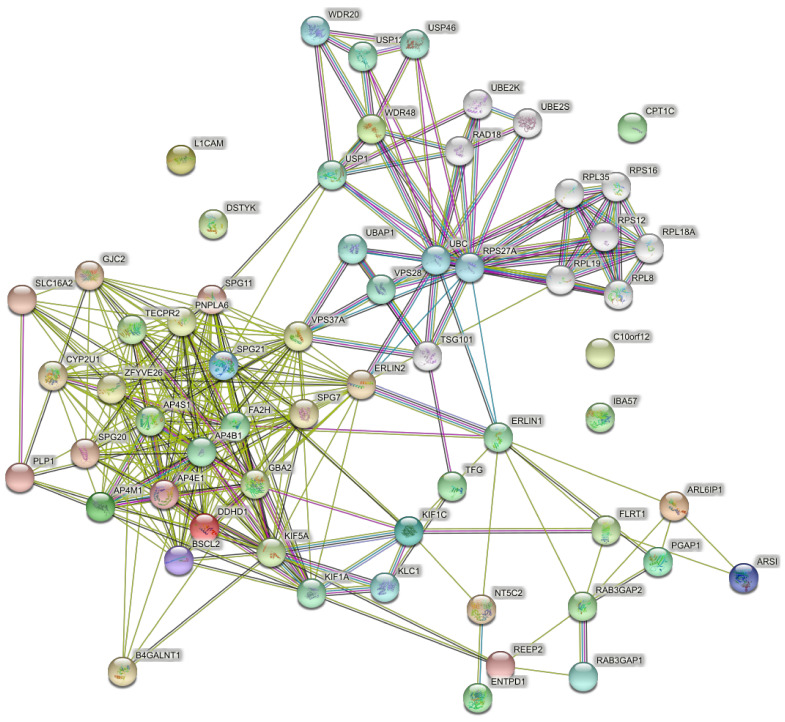
Predicted PPI networks for second shell interactions among 42 genes of complicated SPGs.

For comparison with the 15 pure SPG genes, 42 HSP genes responsible for complicated SPG classifications (Table 1) were used to predict PPI networks. The network of direct interactions between these proteins is shown in Figure 4, with most genes in a highly interconnected central network (PPI enrichment *p* = 1.0 × 10^16^). The second shell revealed the large highly interconnected cluster (bottom left), a ribosomal cluster (top right, RPL genes), and the WASH complex (top, WDR genes), with the latter clusters linked by a highly interconnected ubiquitin cluster (UBC) (Figure 5; PPI enrichment *p* < 1.0 × 10^−16^).

### 4.4. Networks of the Set of Pure and Complicated SPGs (57 Genes)

**Figure 6:**
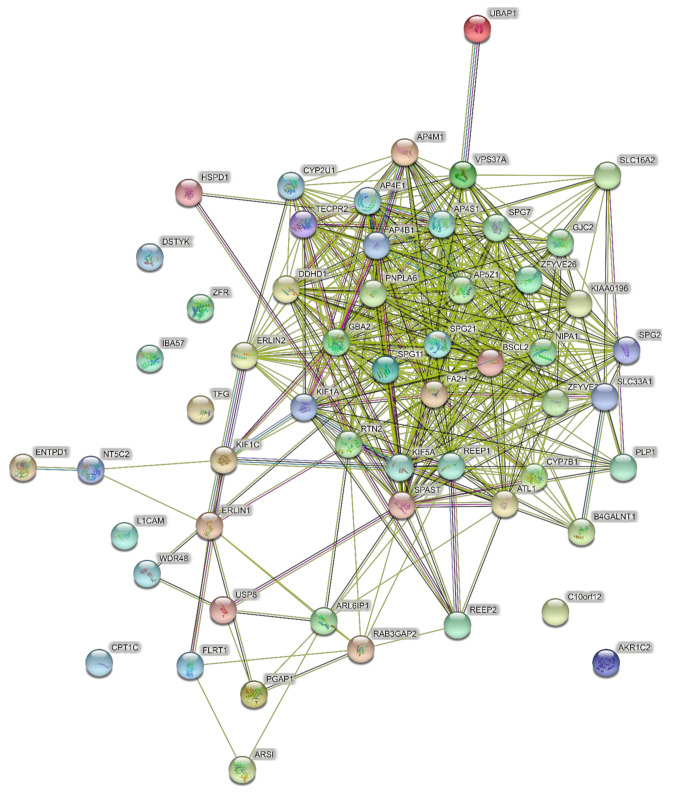
Predicted PPI networks for direct interactions among 57 genes of pure and complicated SPGs.

**Figure 7:**
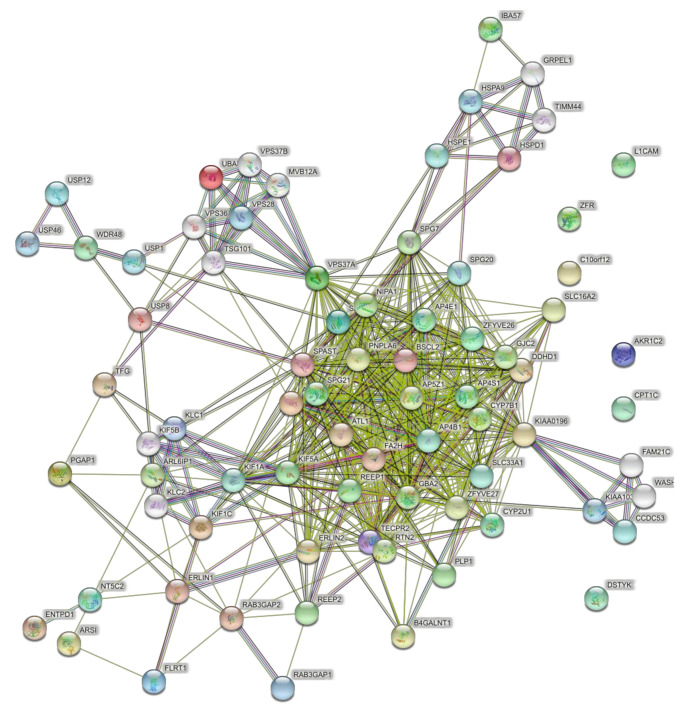
Predicted PPI networks for second shell interactions among 57 genes of pure and complicated SPGs.

When the full set of 57 genes (Table 1) was used including pure and complicated SPG classifications, the network of direct interactions was still highly interconnected (Figure 6; PPI enrichment *p* = 1.0 × 10^−16^). The second shell of interactions was similar to those revealed by the clusters in the second shell interactions of the 15 genes (Figure 7; PPI enrichment *p* < 1.0 × 10^−16^.). Apart from the large central cluster, this PPI network had secondary clusters concerned with the ESCRT I complex (bottom right, VPS genes), the WASH complex (top left, WASH1 gene), and chaperonins (top, HSP genes).

### 4.5. Gene Ontology (GO) Analysis Showed Similar Functions of the PPI Networks in Pure and Complicated HSPs

Table 2 and Table 3 summarize the top most significant GO terms of direct interactions (Table 2) and second shell interactions (Table 3) for the four gene sets.

GO analysis of networks of direct interactions of the six most common pure SPG genes identified endoplasmic reticulum functions as significantly enriched in Biological Processes and Cellular Components (yellow highlighted in Table 2; Supplementary Table S1). GO analysis of the second shell network identified endoplasmic reticulum as well as steroid biochemistry, and the ESCRT III complex was significantly enriched in Biological Processes and Cellular Components (yellow highlighted in Table 3; Supplementary Table S2).

GO analysis of networks of direct interactions of the larger set of 15 pure SPG genes identified was significantly enriched endoplasmic reticulum (as for the six genes) with the addition of localization and transport terms in Biological Processes and Cellular Components (orange highlighted in Table 2; Supplementary Table S3). GO analysis of the second shell network identified transport and localization terms as significantly enriched in Biological Processes (orange highlighted in Table 3) and endoplasmic reticulum and endosome terms were significantly enriched in Cellular Components, the same terms enriched when only six genes were used as input for the network (yellow highlighted in Table 3; Supplementary Table S4).

GO analysis of networks of direct interactions of 42 complicated SPG genes revealed mostly unique GO terms for Biological Processes with no overlap with the pure gene direct PPIs (green highlighted and unhighlighted in Table 2, Supplementary Table S5) whereas there was notable overlap among the Cellular Components dominated by endoplasmic reticulum endoplasmic reticulum, localization, and transport terms in Biological Processes and Cellular Components (yellow highlighted in Table 2). GO analysis of the second shell network identified significant overlap in Biological Processes with the 15 pure gene networks (orange highlighted in Table 3; Supplementary Table S6). Among the Cellular Components, significantly enriched GO terms were dominated by the same endoplasmic reticulum and endosomes GO terms as significantly enriched in pure gene networks (yellow highlighted in Table 3).

GO analysis of networks of direct network of 57 pure and complicated genes identified Biological Processes and Cellular Components that were essentially a mix of those revealed in the pure and complicated gene sets that make up the 57 genes’ analysis (Table 2; Supplementary Table S7). A similar mix was revealed by analysis of the second shell interactions of the 57 genes (Table 3; Supplementary Table S8).

It is notable that the GO Biological Processes and Cellular Components most commonly enriched in all PPI networks were centered on the endoplasmic reticulum, endosomes, and localization, suggesting that mutations in individual HSP genes could have inter-dependent effects on endosome and endoplasmic reticulum structures, functions, and dynamics.

## 5. Future Directions: From Networks to Drug Discovery

Analysis of the PPI networks has revealed some interesting leads for future research aimed at finding new leads for drug discovery for HSP. First, most of the significant Biological Processes and Cellular Components are associated with the endoplasmic reticulum and endosomes, and with transport and localization of organelles, vesicles, proteins, etc. Second, the GO Biological Processes in the direct and second shell PPIs of the 42 complicated genes were different from the 15 pure genes, with a greater contribution from metabolic functions in the complicated gene PPIs. In contrast, the GO Cellular Components were similar between complicated and pure gene PPIs. Third, the direct interactions of just the six common pure genes were enough to identify endoplasmic reticulum functions in the GO Biological Process and Cell components. Fourth, the GO Cellular Components were similar between 15 pure gene PPIs and 42 complicated gene PPIs, indicating that the networks underlying are similar. This similarity of the most significantly networked genes may underlie the clinical signs and symptoms defining HSP. There is less similarity in the GO analyses beyond the top 10 most highly ranked GO categories, which may be explained by gene-specific effects.

The STRING analysis demonstrates that the HSP genes are closely interconnected in PPI networks, affecting a set of related GO Biological Process and Cellular Components that agree with the known associations of HSP genes with different cellular organelles [45]. Many HSP genes have been grouped by association with cellular components and processes of neurons and their axons: mitochondria (SPG7, SPG20, SPG60), Golgi (SPG42), tubulin-dependent organelle transport (SPG4, SPG10, SPG30, SPG31), tubular endoplasmic reticulum (SPG3A, SPG4, SPG12, SPG17, SPG18, SPG31), and endosomes (SPG4, SPG6, SPG8, SPG11, SPG20, SPG31, SPG47, SPG48, SPG50, SPG51, SPG52) [45]. Interestingly, pure and complicated HSP genes are represented in similar components and processes: of the genes above, 10 are from the pure diagnostic classification (SPG3A, SPG4, SPG6, SPG7, SPG8, SPG17, SPG18, SPG31, SPG42, and SPG48) and nine from complicated classification (SPG10, SPG11, SPG12, SPG20, SPG47, SPG50, SPG51, SPG52, and SPG60). The STRING analysis suggests that HSP disease-related functions are regulated by the shared PPI networks of pure and complicated genes. With such highly interconnected PPI networks, it may be possible to alter the network functions shared among the HSP genes with small molecules.

The STRING analysis emphasizes the functions of endoplasmic reticulum and endosomes as new avenues for drug discovery. GO terms associated with endoplasmic reticulum, endosomes, and endomembranes are dominant in the HSP PPI networks. The endoplasmic reticulum is a complex interconnected network of sheets and tubules in which microtubules play a central role in the formation of new tubules and endoplasmic reticulum dynamics [60,61]. The endoplasmic reticulum membrane has specific contacts with peroxisomes, the Golgi apparatus, mitochondria, endosomes, and the plasma membrane [62]. Through these membrane contact sites, the endoplasmic reticulum regulates organelle functions, Ca^2+^ homeostasis, lipid composition, endosome and mitochondrial fission, vesicle trafficking, autophagy, and other important cellular functions [61]. Several HSP genes (*ATL1*/SPG3A, *SPAST*/SPG4, *REEP1*/SPG31, *RTN2*/SPG12) are essential to shaping and maintaining endoplasmic reticulum shape via interactions with the microtubules [60,63]. For example, fission of endosomal tubules is mediated via interaction between spastin and the ESCRT complex [62], which plays a role in the biogenesis of intralumenal membranes of multivesicular bodies [64] as well as dynamics of endolysomes and autophagosomes [64,65,66]. Failure of endosomal tubule fission leads to disruptions of lysosomal morphology and trafficking [67]. Similar lysosomal dysfunction is seen with mutations in *REEP1*/SPG31 and *WASH5C*/SPG8 [67]. Given the central role of endoplasmic reticulum in regulating such a range of cellular functions, it is not unreasonable to hold out hope for new drugs that are effective across several SPG classifications.

The endoplasmic reticulum is the primary site for lipid synthesis and the formation of lipid droplets, which store and transport lipids throughout the cell [66]. Endosomes regulate cholesterol synthesis [66]. GO terms associated with lipid metabolism occur frequently in the STRING analyses including in the top ten ranked by statistical significance for the 15 pure HSP genes (Supplementary Table S2). Mutations in genes affecting lipid metabolic and auxiliary pathway components are associated with multiple motor neuron diseases including Charcot Marie Tooth disease, hereditary motor neuropathy, and amyotrophic lateral sclerosis [68]. HSPs are strongly represented in these pathways: SPG3A, SPG4, SPG5, SPG11, SPG15, SPG17, SPG18, SPG20, SPG26, SPG28, SPG31, SPG35, SPG39, SPG43, SPG46, SPG54, SPG56, and SPG78 [68]. Several HSP genes are involved in lipid droplet formation in the endoplasmic reticulum: *ATL1*/SPG3A, *REEP1*/SPG31 (both at the mitochondrial-ER membrane contact site), *SPAST*/SPG4, *SPG11*, and *ZFYVE26*/SPG15 [68,69]. Spastin plays multiple roles in lipid metabolism at the level of the endoplasmic reticulum where lipids are produced for dispersal through the cell as lipid droplets. Spastin is a positive regulator of lipid metabolism through sorting lipid droplets from the endoplasmic reticulum [70], it coordinates lipid droplet dispersion and endoplasmic reticulum shape along microtubules [71], and tethers lipid droplets to peroxisomes [72].

It is somewhat surprising that mitochondrial function does not feature more strongly in the network analyses. Patient-derived cells from several SPG classifications have mitochondrial dysmorphology, lowered mitochondrial membrane potential, altered transport and reduced oxidative phosphorylation (see above), exemplified by cells from people with *SPG7*/paraplegin [41]. It seems, therefore, that mitochondrial dysfunction is not a shared direct effect of these HSP gene PPI networks. Mitochondrial function may be altered in other SPGs, but downstream of direct regulation by these networks.

There are already a few candidate small molecules that show promise as leads in HSP treatment. These include tubulin-binding drugs (taxol, vinblastine, epothilone D, noscapine), a liver X receptor (LXR) agonist (GW3965), a GSK3 inhibitor (Tidegluib), and an inhibitor of mitochondrial Complex I (mdvi-1). None of these is known to specifically target endoplasmic and endosome functions, but some are already shown to be active in more than one SPG classification.

Low doses of tubulin-binding drugs increase stable microtubule levels (acetylated α-tubulin) in SPAST HSP patient cells to the levels in healthy control cells [28,33,73]. By restoring the numbers of stable microtubules, these drugs reversed the impairment in peroxisome transport and ameliorated the oxidative stress in SPG4/*SPAST* patient-derived olfactory neural stem cells and patient-derived neurons [28,33,74]. Tubulin-binding drugs vinblastine and taxol also rescued the axon growth deficits in SPG3A/*SPG3A* iPS cell-derived patient neurons [46]. Noscapine and epothilone D are potential new treatments because they can pass the blood–brain barrier. Noscapine is a good candidate because it is approved in some countries for other indications, it is off-patent, and could be repurposed for SPG3A and SPG4 HSP.

GW3965, a liver X receptor (LXR) agonist, rescued three phenotypes (reduced neurite growth, increased growth cone area, axonal swellings) in SPAST HSP neurons, providing a potential drug target for HSP treatment [36]. Activation of LXR upregulates cholesterol synthesis and cholesterol efflux out of the cells [75] and GW3965 modulates cholesterol metabolism and transport, lipogenesis, and protection from cholesterol over-load [76]. Endogenous agonists of LXR include 27-hydroxycholesterol and 25-hydroxycholesterol [47] whose plasma and cerebrospinal fluid levels are highly raised in SPG5 HSP patients [50]. SPG5 encodes the *CYP7B1* gene, a paralog of *CYP7A1*, both of which are involved in cholesterol, metabolism [47]. CYP7B1 converts 27-hydroxycholesterol into 5-cholesten-3β,7α,27-triol [48]. Mutations that block the activity of CYP7B1 would be expected to raise plasma levels 27-hydroxycholesterol, which is excreted via LXR-dependent mechanisms. Cholesterol lowering statins have been suggested as a potential therapeutic in SPG5 [50].

Tideglusib, a GSK3 inhibitor, restored neurite number and length in SPG11 iPS cell derived patient neurons [53]. GSK3B is a regulatory protein kinase involved in many cell pathways including cholesterol synthesis and microtubule stabilization [77]. Widely used statins that inhibit cholesterol metabolism inhibit microtubule-associated Tau accumulation in neurons derived from Alzheimer’s disease iPS cells [74]. With loss of spatacsin in SPG11 patient-derived fibroblasts, microtubule formation is inhibited, leading to impaired export from late endosomes/lysosomes and causing them to accumulate cholesterol [78]. Thus, the SPG11 mutation directly links microtubule stabilization, cholesterol synthesis, and microtubule-dependent organelle transport.

Treatment with mdvi-1 inhibits basal and maximal respiration at mitochondrial complex I via inhibition of the dynamin-related protein-1 (Drp1), which reduces mitochondrial fission [79]. In SPG15 and SPG48 iPS cell derived patient neurons, mdvi-1 treatment rescued mitochondrial morphology and neurite outgrowth [55].

New drug candidates under investigation for other diseases may also prove useful for HSP and should be considered for further research. Metformin, a widely used diabetes drug, promotes axon regeneration after spinal cord injury in rat [80]. It does this by stabilizing microtubules and may be a substitute for noscapine in SPG4. Trichostatin A ameliorated axon degeneration in the SOD1 mouse model of ALS [81]. Trichostatin A increases stabilized microtubules by inhibiting deacetylation. Riluzole improved outcomes in a clinical trial in ALS [82], potentially by stabilizing microtubule turnover, as shown in the SOD1 mouse model [83]. Troriluzole, a riluzole pro-drug, is being trialed for spinocerebellar ataxia [84]. Pre-084, an agonist of the SIGMAR1 receptor, prevented axon degeneration in cultured mouse motor neurons [85]. Mutations in SIGMAR1 are associated with distal hereditary motor neuropathy [86]. SIGMAR1 is localized on the endoplasmic reticulum outer membrane at points of contact with mitochondria [87] where it regulates mitochondrial calcium homeostasis and ER stress activation [85]. Liraglutide, approved for use in diabetes, is neuroprotective through modulation of the ER stress response, shifting cell fate from apoptosis to survival in a neuroblastoma cell line [88]. Dantrolene, an endoplasmic reticulum calcium channel blocker approved for diabetes, reduces ER stress through its action on endoplasmic reticulum Ca^2+^ dynamics [89]. These examples provide hope that among the many approved drugs already in clinical use, there will be some that are effective for HSP, perhaps by acting on endoplasmic reticulum functions, vesicle trafficking, and interactions with microtubules and other organelles.

Already, we know that the effects of *SPAST* mutations in patient-derived neurons can be reversed by drugs with entirely different mechanisms of action, namely, tubulin binding and LXR receptor agonist drugs, with mechanisms of action targeting cholesterol metabolism [33,36]. On the other hand, neurite outgrowth and axon dysmorphology are cell phenotypes shared by patient-derived cells representing mutations across several SPG classifications (see above). The connection between these mechanisms may help elucidate cell pathways or networks shared across the HSP gene spectrum. Another significant observation is that SPG4, SPG5, and SPG11 cell pathologies are linked via the LXR pathway and that SPG4 and SPG11 have shared pathologies in microtubule stabilization, organelle transport, and cholesterol metabolism.

The STRING analysis is an underestimate of the full extent of “HSP PPI networks” because the STRING database is limited to, and biased by, published data so that genes of greater research interest (e.g., cancer genes) are better annotated and have more information about them. These PPI networks are based on predicted protein–protein interactions but do not take into account the consequences of the interactions (such as phosphorylation and its downstream consequences) or the enzymatic functions that might ensue (for example, severing of stable microtubules by spastin). Consequently, the networks here under-represent the full extent of the roles of these proteins in cellular functions. Nonetheless, the networks place the HSP proteins in cellular locations and in cell functions, which can become the basis for drug discovery based on cell phenotype.

One of the consequences of highly interconnected gene networks is that mutation of an individual gene affects network function. Although mutations affect individual genes and their molecular functions, mutations in different genes can lead to similar cell phenotypes through the interactions in a network and subsequent homeostatic compensation [28]. Treatments that modulate network functions may not need to target a particular genotype when the aim is to moderate shared cell phenotypes from multiple genotypes. In this paradigm, drug discovery is based on patient-derived cell phenotypes, taking into account all the variability that entails, focusing away from cellular and molecular mechanisms of mutations in individual genes.

For a drug discovery program, it would be ideal to screen patient-derived cells from multiple SPG classifications using a standardized set of cell functions. ONS cells are a useful screening tool because they can be grown at scale in standardized conditions for HSP research [28,41,73]. This network analysis suggests some novel cell functions to explore (e.g., endoplasmic stress, endoplasmic reticulum morphology, or other endoplasmic reticulum functions) that could be developed for standardized high throughput assays across different genotypes in patient-derived cells. These functional phenotypes would be used for screening compound libraries to identify new drug leads.

## Figures and Tables

**Table 1:** Clinical SPG classifications and HSP genes.

Clinical SPG Classifications and HSP Genes					
HSP	Inheritance	Gene Name	HSP	Inheritance	Gene Name
Classification			Classification		
SPG01	XL	L1CAM *	SPG41	AD	Gene Locus
SPG02	XL	PLP1 *	SPG42	AD	SLC33A1 **
SPG03A	AD	ATL1 ***	SPG43	AR	C10orf12 *
SPG04	AD	SPAST ***	SPG44	AR	GJC2 *
SPG05A	AR	CYP7B1***	SPG45	AR	NT5C2 *
SPG06	AD	NIPA1 **	SPG46	AR	GBA2 *
SPG07	AR	SPG7 *	SPG47	AR	AP4B1 *
SPG08	AD	WASHC5 **	SPG48	AR	AP5Z1 **
SPG09	AR	ALDH18A1	SPG49	AR	TECPR2 *
SPG10	AD	KIF5A *	SPG50	AR	AP4M1 *
SPG11	AR	SPG11 *	SPG51	AR	AP4E1 *
SPG12	AD	RTN2***	SPG52	AR	AP4S1 *
SPG13	AD	HSPD1 **	SPG53	AR	VPS37A *
SPG14	AR	Gene Locus	SPG54	AR	DDH2 **
SPG15	AR	ZFYVE26 *	SPG55	AR	C19ORF65R
SPG16	XL	Gene Locus	SPG56	AR	CYP2U1 *
SPG17	AD	BSCL2 *	SPG57	AR	TFG *
SPG18	AR	ERLIN2 *	SPG58	AR	KIF1C *
SPG19	AD	Gene Locus	SPG59	AR	USP8 **
SPG20	AR	SPART *	SPG60	AR	WDR48 *
SPG21	AR	SPG21 *	SPG61	EX	ARL6IP1 *
SPG22	XL	SLC16A2 *	SPG62	AR	ERLIN1 *
SPG23	AR	DSTYK *	SPG63	AR	AMPD2
SPG24	AR	Gene Locus	SPG64	AR	ENTPD1 *
SPG25	AR	Gene Locus	SPG65	AR	NT5C2 *
SPG26	AR	B4GALNT1* *	SPG66	AR	ARSI *
SPG27	AR	Gene Locus	SPG67	AR	PGAP1 *
SPG28	AR	DDHD1 *	SPG68	AR	FLRT1 *
SPG29	AD	Gene Locus	SPG69	AR	RAB3GAP2 *
SPG30	AR	KIF1A *	SPG70	AR	MARS1
SPG31	AD	REEP1 ***	SPG71	AR	ZFR **
SPG32	AD	Gene Locus	SPG72	AR	REEP2 *
SPG33	AD	ZFYVE27 ***	SPG73	AD	CPT1C *
SPG34	XL	SPG34	SPG74	AR	IBA57 *
SPG35	AR	FA2H *	SPG75	AR	MAG
SPG36	AD	SPG36	SPG76	AR	CAPN1
SPG37	AD	SPG37	SPG77	AR	FARS2
SPG38	AD	SPG38	SPG78	AR	ATP13A
SPG39	AR	PNPLA6 *	SPG79	AR	UCHL1
SPG40	AD	Gene Locus	SPG80	AD	UBAP1 **

Notes to Table 1: * STRING Analysis of 42 genes from COMPLICATED SPGs. ** STRING Analysis of 15 genes from PURE SPGs. *** STRING Analysis of six genes from MOST COMMON PURE SPGs. Source references: [2,3,4,5,10,11,12,13]; Genecards (genecards.org; accessed on 25 January 2021), OMIM (omim.org; accessed on 25 January 2021).

**Table 2:** Gene Ontology (GO) terms of protein–protein interactions (PPIs) of direct interactions (top ranked by false discovery rate, FDR).

6 Most Common Pure SPGs	15 Pure SPGs	42 Complicated SPGs	57 Pure and Uncomplicated SPGs
**Biological process**			
endoplasmic reticulum tubular network organization	endoplasmic reticulum tubular network organization	retrograde neuronal dense core vesicle transport	endoplasmic reticulum tubular network
endomembrane system organization	endomembrane system organization	intracellular transport	intracellular transport
	cytoskeleton-dependent cytokinesis	axo-dendritic transport	cellular localization
	cellular localization	lipid metabolic process	amide transport
	establishment of localization	intracellular protein transport	axo-dendritic transport
	localization	negative regulation of cholesterol biosynthetic process	endomembrane system organization
	protein localization	anterograde neuronal dense core vesicle transport	establishment of protein localization
	endosomal transport	DREBP signaling pathway	localization
	intracellular transport	cytosolic transport	establishment of localization
	amide transport	establishment of vesicle location	retrograde neuronal dense core vesicle transport
**Cellular Component**			
endoplasmic reticulum tubular network	endoplasmic reticulum tubular network	organelle subcompartment	organelle subcompartment
endoplasmic reticulum membrane	endosome	AP4-adaptor complex	endoplasmic reticulum membrane
endoplasmic reticulum subcompartment	endomembrane system	organelle membrane	endoplasmic reticulum subcompartment
axon	endoplasmic reticulum membrane	endoplasmic reticulum membrane	organelle membrane
integral component of endoplasmic reticulum membrane	endoplasmic reticulum subcompartment	endoplasmic reticulum subcompartment	endomembrane system
	endoplasmic reticulum	endomembrane system	endoplasmic reticulum
	organelle membrane	endoplasmic reticulum	AP-4 adaptor complex
	early endosome	endosome lumen	endosome
	cytoplasm	trans-Golgi network membrane	AP-type membrane coat adaptor complex
	neuron projection	membrane	endoplasmic reticulum tubular network

Note: Colors represent GO terms shared among the PPIs.

**Table 3:** GO terms of PPIs of second shell interactions (top ranked by FDR).

6 Most Common Pure SPGs	15 Pure SPGs	42 Complicated SPGs	57 Pure and Uncomplicated SPGs
**Biological Process**			
multivesicular body assembly	endosomal transport	organic substance catabolic process	intracellular transport
viral budding	amide transport	intracellular transport	establishment of protein localization
endomembrane system organization	establishment of protein localization	catabolic process	amide transport
steroid biosynthesis process	protein transport	cellular macromolecule catabolic process	protein transport
septum digestion after cytokinesis	intracellular transport	cellular catabolic process	cellular localization
viral budding via host ESCRT complex	protein localization	protein targeting	establishment of localization
viral life cycle	virion assembly	establishment of protein localization	protein localization
ESCRT III complex disassembly	cellular localization	intracellular protein transport	macromolecular localization
androgen biosynthetic process	organic substance transport	establishment of protein localization to endoplasmic reticulum	endomembrane system organization
vacuolar transport	endosome organization	protein transport	localization
**Cellular Component**			
organelle membrane	endoplasmic reticulum tubular network	organelle subcompartment	organelle subcompartment
ESCRT III	endosome	AP4-adaptor complex	endoplasmic reticulum membrane
endomembrane system	endomembrane system	organelle membrane	endoplasmic reticulum subcompartment
endoplasmic reticulum membrane	endoplasmic reticulum membrane	endoplasmic reticulum membrane	organelle membrane
endoplasmic reticulum subcompartment	endoplasmic reticulum subcompartment	endoplasmic reticulum subcompartment	endomembrane system
endosome membrane	endoplasmic reticulum	endomembrane system	endoplasmic reticulum
late endosome membrane	organelle membrane	endoplasmic reticulum	AP-4 adaptor complex
endoplasmic reticulum tubular network	early endosome	endosome lumen	endosome
endosome	cytoplasm	trans-Golgi network membrane	AP-type membrane coat adaptor complex
endoplasmic reticulum	neuron projection	membrane	endoplasmic reticulum tubular network

Note: Colors represent GO terms shared among the PPIs.

## Supplementary Materials

The following are available online at https://www.mdpi.com/2076-3425/11/3/403/s1, Table S1: Gene Ontology (GO) analysis of six common pure gene direct network, ranked by false discovery rate (FDR); Table S2: GO analysis of six common pure hereditary spastic paraplegias (HSP) gene second shell network, ranked by FDR; Table S3: GO analysis of 15 pure HSP gene direct network, ranked by FDR; Table S4: GO analysis of 15 pure HSP gene second shell network, ranked by FDR; Table S5: GO analysis of 42 complicated HSP gene direct network, ranked by FDR; Table S6: GO analysis of 42 complicated HSP gene second shell network, ranked by FDR; Table S7: GO analysis of 57 pure and complicated HSP gene direct network, ranked by FDR; Table S8: GO analysis of 57 pure and complicated HSP gene second shell network, ranked by FDR.
